# Pembrolizumab-axitinib versus nivolumab-cabozantinib as first-line therapy in patients with metastatic renal cell carcinoma: a retrospective real-world comparison (ARON-1)

**DOI:** 10.1007/s00262-025-04043-x

**Published:** 2025-05-27

**Authors:** Matteo Santoni, Giandomenico Roviello, Enrique Grande, Ugo De Giorgi, Ondrej Fiala, Emmanuel Seront, Javier Molina-Cerrillo, Renate Pichler, Zin W. Myint, Jakub Kucharz, Ravindran Kanesvaran, Thomas Büttner, Martin Pichler, Umberto Basso, Jindrich Kopecky, Maria T. Bourlon, Linda Cerbone, Tomas Buchler, Alvaro Pinto, Alfonso Gómez de Liaño, Caterina Gianni, Anca Zgura, Pasquale Rescigno, Jawaher Ansari, Orazio Caffo, Zsófia Küronya, Maria Giuseppa Vitale, Dipen Bhuva, Martina Catalano, Nuno Vau, Ray Manneh Kopp, Sebastiano Buti, Aristotelis Bamias, Camillo Porta, Kaisa Sunela, Francesco Massari

**Affiliations:** 1 Oncology Unit, Macerata Hospital, Macerata, Italy; 2 Department of Health Sciences, Section of Clinical Pharmacology and Oncology, University of Florence, Viale Pieraccini 6, 50139 Florence, Italy; 3 Department of Medical Oncology, MD Anderson Cancer Center Madrid, Madrid, Spain; 4 Department of Medical Oncology, IRCCS Istituto Romagnolo Per Lo Studio Dei Tumori (IRST) “Dino Amadori”, Meldola, Italy; 5 Department of Oncology and Radiotherapeutics, Faculty of Medicine and University Hospital in Pilsen, Charles University, Pilsen, Czech Republic; 6 Department of Medical Oncology, Cliniques Universitaires Saint-Luc 1200, Brussels, Belgium; 7 Hospital Ramón y Cajal, Madrid, Spain; 8 Department of Urology, Medical University of Innsbruck, Anichstrasse 35, 6020 Innsbruck, Austria; 9 Markey Cancer Center, University of Kentucky, Lexington, KY 40536-0293 USA; 10 Department of Uro-Oncology, Maria Sklodowska-Curie National Research Institute of Oncology Warsaw, Warsaw, Poland; 11 Division of Medical Oncology, National Cancer Centre Singapore, Singapore, Singapore; 12 Department of Urology, University Hospital Bonn (UKB), 53127 Bonn, Germany; 13 Division of Oncology, Department of Internal Medicine, Medical University of Graz, Augenbruggerplatz 15, 8010 Graz, Austria; 14 Oncology 3 Unit, Department of Oncology, Istituto Oncologico Veneto IOV IRCCS, Padua, Italy; 15 Department of Clinical Oncology and Radiotherapy, University Hospital Hradec Kralove, Hradec Kralove, Czechia; 16 Hematology and Oncology Department, Instituto Nacional de Ciencias Médicas y Nutrición Salvador Zubirán, Mexico City, Mexico; 17 Department of Medical Oncology, San Camillo Forlanini Hospital, Rome, Italy; 18 Department of Oncology, Second Faculty of Medicine, Charles University and Motol University Hospital Prague, Prague, Czech Republic; 19 Medical Oncology Department, La Paz University Hospital, Madrid, Spain; 20 Medical Oncology Department, CHU Insular-Materno Infantil, Las Palmas de Gran Canaria, Spain; 21 Department of Obstetrics-Radiotherapy, Alexandru Trestioreanu Institute of Oncology, “Carol Davila” University of Medicine and Pharmacy, Prof. Dr, Bucharest, Romania; 22 Translational and Clinical Research Institute, Centre for Cancer, Newcastle University, Newcastle Upon Tyne, UK; 23 Medical Oncology, Tawam Hospital, Al Ain, United Arab Emirates; 24 Medical Oncology Unit, Santa Chiara Hospital, Trento, Italy; 25 Department of Genitourinary Medical Oncology and Clinical Pharmacology, National Institute of Oncology, Budapest, Hungary; 26 Department of Oncology and Hematology, University Hospital of Modena, Modena, Italy; 27 Department of Medical Oncology, Army Hospital Research and Referral, New Delhi, India; 28 Urologic Oncology, Champalimaud Clinical Center, 1400-038 Lisbon, Portugal; 29 Clinical Oncology, Sociedad de Oncología y Hematología del Cesar, Valledupar, Colombia; 30 Medical Oncology Unit, University Hospital of Parma, 43126 Parma, Italy; 31 2nd Propaedeutic Department of Internal Medicine, School of Medicine, ATTIKON University Hospital, National and Kapodistrian University of Athens, Athens, Greece; 32 Interdisciplinary Department of Medicina, University of Bari “Aldo Moro” and Division of Medical Oncology, A.O.U. Consorziale Policlinico Di Bari, Bari, Italy; 33 Finnish Medicines Agency Fimea, Tampere, Finland; 34 Medical Oncology, IRCCS Azienda Ospedaliero-Universitaria Di Bologna, Via Albertoni, 15, Bologna, Italy

**Keywords:** ARON-1 study, Axitinib plus pembrolizumab, Cabozantinib plus nivolumab, Immune-oncology combinations

## Abstract

**Background:**

The optimal first-line therapy for metastatic renal cell carcinoma (mRCC) remains uncertain, despite recent advancements in immune-based combinations. This retrospective study compares the effectiveness of pembrolizumab plus axitinib (PA) and nivolumab plus cabozantinib (NC) as first-line treatments for mRCC in a real-world setting.

**Methods:**

Patient data were collected from 55 centers across 16 countries, encompassing individuals diagnosed with mRCC receiving first-line treatment with PA or NC between January 2016 and October 2023. Clinical and tumor features and treatment responses were recorded. The primary endpoints were overall response rate (ORR), overall survival (OS), progression-free survival (PFS), and time to second progression. Statistical analyses included Kaplan–Meier survival estimates, Cox proportional hazard models, and chi-square tests.

**Results:**

A total of 760 patients with a median age of 64 years (range, 29–88) were included. Of them, 607 received PA, and only 153 NC. In the overall study population, ORR was 59% for and 49% for PA. Median OS was 55.7 months and not reached (NR) for PA and NC, respectively (*P* = .51), while median PFS was longer with NC (27.6 months) than for PA (16.2 months, *P* = .003). Subgroup analysis suggested a PFS benefits for NC in male, younger patients, intermediate risk group, clear cell histology, and lung involvement, as well as ORR favored NC in good risk patients. Multivariate analysis identified first-line therapy as a significant factor associated with PFS.

**Conclusions:**

In this certainly biased retrospective comparison, NC demonstrated superior ORR and longer PFS compared to PA in mRCC. These findings underscore the importance of considering individual patient characteristics and risk profiles when selecting first-line therapy for mRCC.

**Supplementary Information:**

The online version contains supplementary material available at 10.1007/s00262-025-04043-x.

## Introduction

Renal cell carcinoma (RCC) comprises 90–95% of kidney cancers and 3% of all adult cancers [1]. Among RCC histological subtypes, clear cell cancer histology is the most common, accounting for 75% of them, followed by papillary, chromophobe, and a number of rarer entities [2]. RCC is typically diagnosed as localized disease, with approximately 25% of cases relapsing after the radical resection of the primary tumor, while one-third of patients already have metastatic disease at diagnosis [3]. Despite the diagnostic and therapeutic progresses achieved over the past decade, RCC remains one of the most deadly urological malignancies [1].

Since the advent of cytokines, patients with metastatic RCC have been categorized into three prognostic groups based on clinical and laboratory features: favorable, intermediate, and poor. Currently, the Memorial Sloan Kettering Cancer Center (MSKCC) and International mRCC Database Consortium (IMDC) models remain the predominant prognostic tools used in clinical practice [4, 5].

In the last few years, a number of phase III randomized clinical trials, utilizing sunitinib as the comparator arm, have established a novel standard of care incorporating immune checkpoint inhibitors (ICIs) in the management of advanced RCC [6–11]. Although there have been notable advances in treatment with the introduction of immunotherapy, patient stratification remains an essential component of clinical decision-making and treatment selection for this patient population.

Notably, pembrolizumab plus axitinib (PA) (Keynote-426), nivolumab plus cabozantinib (NC) (CheckMate 9ER), pembrolizumab plus lenvatinib (CLEAR), and avelumab plus axitinib (JAVELIN Renal 101) have received approval though with significant regional variations, irrespective of the IMDC risk group [6–9]. However, nivolumab plus ipilimumab (CheckMate 214) has only been approved for patients with intermediate/poor risk according to the IMDC criteria [10].

With such a wide range of different treatment combinations now available, comparative analyses of their effectiveness and safety would be needed for guiding optimal first-line therapy selection. On this scenario, the ARON-1 study (NCT05287464) was designed to globally collect real-world data on the use of immune combinations as first-line therapy for mRCC [12–15]. In this sub-analysis, we conducted a retrospective comparison of the effectiveness of PA *versus* NC as first-line therapy in patients with advanced RCC.

## Patients and methods

### Study population

We retrospectively collected data from patients aged ≥ 18 years with a histologically confirmed diagnosis of RCC and histologically or radiologically confirmed metastatic disease. We included patients treated with first-line PA or NC between January 1st 2016 to October 1st 2023 from 55 centers from 16 countries.

We retrospectively extracted from patients’ paper and electronic charts data about age, gender, tumor histology, nephrectomy, sites of metastases, type of immuno-combination and response to therapy. Patients with insufficient data on tumor assessment or response to therapy were excluded from the ARON-1 study.

First-line therapy was continued till the evidence of clinical and/or radiological tumor progression, unacceptable toxicities, or death. Computed tomography or magnetic resonance imaging scans were performed following standard local procedures every 8–12 weeks. Physical examination and laboratory tests were carried out every 4–6 weeks during patients’ follow-up.

### Study endpoints

The primary objective of our retrospective study was to assess the outcome of patients treated with first-line PA or NC for advanced RCC. Tumor radiological assessment was led according to the RECIST 1.1 criteria [16] and data on tumor response (complete [CR] or partial responses [PR], stable [SD] or progressive disease [PD]) were collected and analyzed. Overall Response Rate (ORR) was defined as the proportion of patients who achieve a CR or PR per RECIST criteria. Overall Survival (OS) was calculated from the start of treatment to death for any cause. Progression-Free Survival (PFS) was defined as the time from the start of first-line therapy to progression or death from any cause. Time to second progression (PFS2) was defined as the time from the start of first-line therapy to objective tumor progression on next-line treatment or death from any cause. Patients without a tumor progression to following line of treatment or death or lost at follow-up at the time of analysis were censored at their last follow-up date.

### Statistical analysis

OS, PFS and PFS2 were estimated using the Kaplan–Meier method with Rothman’s 95% confidence intervals (CI), and comparisons between survival distributions were led by using the log-rank test. Univariate and multivariate analyses were carried out by using Cox proportional hazard models, Hazard Ratio (HR) and their 95% confidence intervals (95%CI). The chi-square test was employed to compare groups for categorical variables. Significance levels were set at a value of 0.05, and all *p* values were two-sided. The statistical analysis was performed by MedCalc version 19.6.4 (MedCalc Software, Broekstraat 52, 9030 Mariakerke, Belgium).

## Results

### Study population

In the ARON-1 study, we analyzed data relative to 3902 mRCC patients. Of them, 760 (19%) were included in this analysis (Fig. S1). The median follow-up time was 17.0 months (95%CI 15.9−68.1); 557 patients (73%) were males. Median age was 64 years (range 29−88). Tumor histology was clear cell RCC in 643 patients (85%); Sarcomatoid feature was reported in 87 patients (11%). Most patients underwent a nephrectomy (63%). The most frequent sites of metastases were lungs (64%) and bones (36%).

Six hundred and seven patients (80%) received PA as first-line therapy while 153 patients (20%) were treated with the NC combination, due to the different timing of availability of the two options; 184 patients (24%) had died at the time of analysis. Baseline clinical and pathological characteristics of the overall population are shown in Table 1. Notably, patients receiving NC were enriched for poor prognostic features such as IMDC risk classification and presence of bone and liver metastasis, without significant difference.

**Table 1:** Baseline patients’ characteristics

Patients	Overall 760 (%)	Pembrolizumab + Axitinib 607 (%)	Nivolumab + Cabozantinib 153 (%)	*P*
Gender				
Male	557 (73)	447 (74)	110 (72)	.75
Female	203 (27)	160 (26)	43 (28)	
Age, years (y)	64	65	64	–
Range	29−88	34−88	29−87	
Metastatic at diagnosis	415 (55)	338 (56)	77 (50)	.48
Prior nephrectomy	477 (63)	382 (63)	95 (62)	.88
Clear cell histology	643 (85)	513 (85)	130 (85)	> .99
Sarcomatoid feature	87 (11)	67 (11)	20 (13)	.66
IMDC risk stratification				
Favorable risk	163 (22)	130 (21)	33 (22)	.21
Intermediate risk	413 (55)	343 (57)	70 (46)	
Poor risk	184 (23)	134 (22)	50 (32)	
Common sites of metastasis				
Lung	483 (64)	384 (63)	99 (65)	.76
Bone	271 (36)	203 (33)	68 (44)	.11
Liver	139 (18)	102 (17)	37 (24)	.22
Brain	57 (8)	47 (8)	7 (5)	.39
Median follow-up	17.0 months	18.3 months	15.1 months	.38
Second-line therapies	224 (29)	206 (34)	18 (12)	< .001
Type of second-line therapy				
Cabozantinib	191 (25)	191 (31)	0 (0)	–
Sunitinib	15 (2)	0 (0)	15 (10)	
Clinical Trials	7 (1)	4 (1)	3 (2)	
Other	11 (1)	11 (2)	0 (0)	

*IMDC* International Metastatic RCC Database Consortium

### Survival analysis

**Fig. 1:**
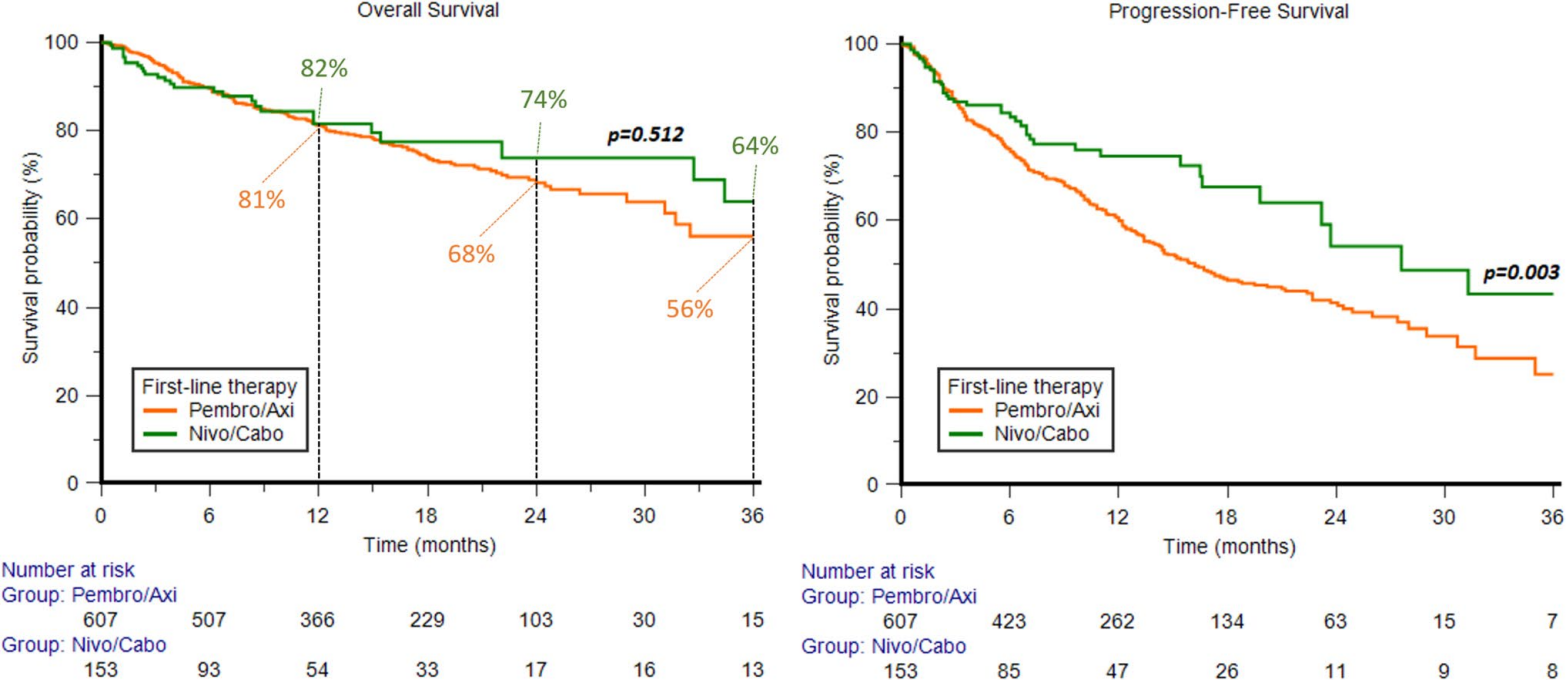
Overall survival and progression-free survival among metastatic renal cell carcinoma patients

In the overall study population, the median OS and PFS were 52.2 months (95%CI 34.4−55.7) and 17.6 months (95%CI 15.4−22.7), respectively. The median OS was 55.7 months (95%CI 31.7−55.7) in patients receiving PA and not reached (NR) (95%CI NR−NR) in patients receiving NC (*P* = 0.51, Fig. 1). The 1y-, 2y- and 3y-OS rates were 81% *vs.* 82% (*P* = 0.85), 68% *vs*. 74% (*P* = 0.61) and 56% *vs*. 64% (*P* = 0.24) for PA *vs.* NC, respectively.

Median PFS, it was 16.2 months (95%CI 14.2−20.2) in patients receiving PA and 27.6 months (95%CI 23.2−51.5) in patients receiving NC (*P* = 0.003, Fig. 1). The 1y-, 2y- and 3y-PFS rates were 62% vs 77% (*P* = 0.02), 43% *vs.* 56% (*P* = 0.06) and 29% *vs*. 45% (*P* = 0.01) for PA *vs.* NC, respectively.

**Fig. 2:**
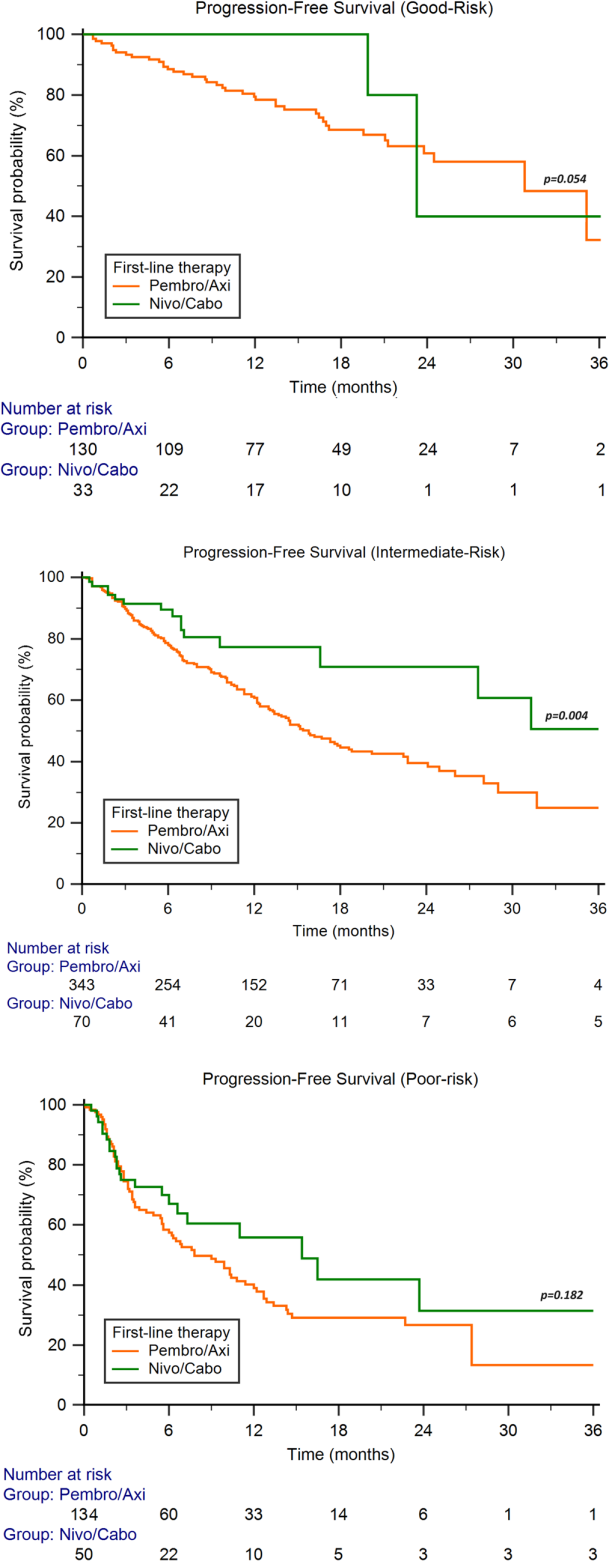
Progression-free survival according to International Metastatic RCC Database Consortium score

In patients with good risk features, the median OS was NR in both subgroups (*P* = 0.15), while the median PFS was 30.7 months (95%CI 23.7−35.0) for PA and NR (95%CI NR−NR) for NC (*P* = 0.05). In the intermediate risk subgroup, the median OS was 40.5 months (95%CI 29.0−55.7) for PA and NR (95%CI NR−NR) for NC (*P* = 0.11), with a significantly longer median PFS (31.3 months, 95%CI 27.6−40.6 vs 15.2 months, 95%CI 13.3−53.3, *P* = 0.004) in patients receiving NC. Finally, poor risk patients showed a median OS and PFS of 15.5 months (95%CI 12.4−22.1) and 7.8 months (95%CI 5.6−11.4) in those receiving PA and 22.1 months (95%CI 11.7−32.7, *P* = 0.86) and 15.4 months (95%CI 6.6−51.5, *P* = 0.18) in patients treated with NC (Fig. 2).

We further stratified patients based on tumor histology. No significant differences were found in terms of OS in both patients with clear cell (PA: 55.7 months, 95%CI 55.7−55.7; NC: NR, 95%CI NR−NR, *P* = 0.74) and non-clear cell RCC histology (PA: 31.1 months, 95%CI 24.5−40.5; NC: 24.9 months, 95%CI 14.9−34.9, *P* = 0.98). On the other hand, we observed longer median PFS in patients with clear cell RCC histology treated with NC (31.3 months, 95%CI 23.2−51.5) compared to PA (17.1 months, 95%CI 23.2−51.5, *P* = 0.005, Fig. S2) while no significant differences were find in terms of median PFS (PA: 12.3 months, 95%CI 10.0–15.8; NC: 15.4 months, 95%CI 6.9–18.9; *P* = 0.569).

In the 87 patients with sarcomatoid feature, the median OS and PFS were NR (95%CI NR−NR) and 11.4 months (95%CI 4.1−28.0) for PA and 25.3 months (95%CI 8.3−34.4, *P* = 0.69) and 23.2 months (95%CI 5.5−23.2, *P* = 0.73) for NC.

We further stratified patients by site of metastasis, showing no differences in terms of median OS between patients treated by PA and NC with lung (NR, 95%CI NR−NR, *vs.* NR, 95%CI NR−NR, *P* = 0.37), liver (55.7 months, 95%CI 19.5−55.7, *vs.* 32.7 months, 95%CI 15.4−52.2, *P* = 0.86), or bone (29.0 months, 95%CI 23.6−55.7, *vs*. 22.1 months, 95%CI 14.9−40.0, *P* = 0.26) or brain metastases (29.0 months, 95%CI 15.4−40.5, *vs*. 11.7 months, 95%CI 1.3−11.7, *P* = 0.64).

Median PFS, was significantly longer in patients with lung metastases treated with NC (15.4 months, 95%CI 13.4−18.0, *vs.* 40.6 months, 95%CI 16.6−51.5, *P* = 0.003, Fig. S2), while no statistically significant differences were found in patients with bone (12.0 months, 95%CI 9.9−14.5, *vs*. 16.5 months, 95%CI 11.0−16.6, *P* = 0.19), liver (12.2 months, 95%CI 6.2−17.3, *vs*. 16.5 months, 95%CI 6.6−27.6, *P* = 0.38) or brain metastases (9.9 months, 95%CI 7.0−30.7 *vs*. 6.9 months, 95%CI 1.3−6.9, *P* = 0.78).

**Fig. 3:**
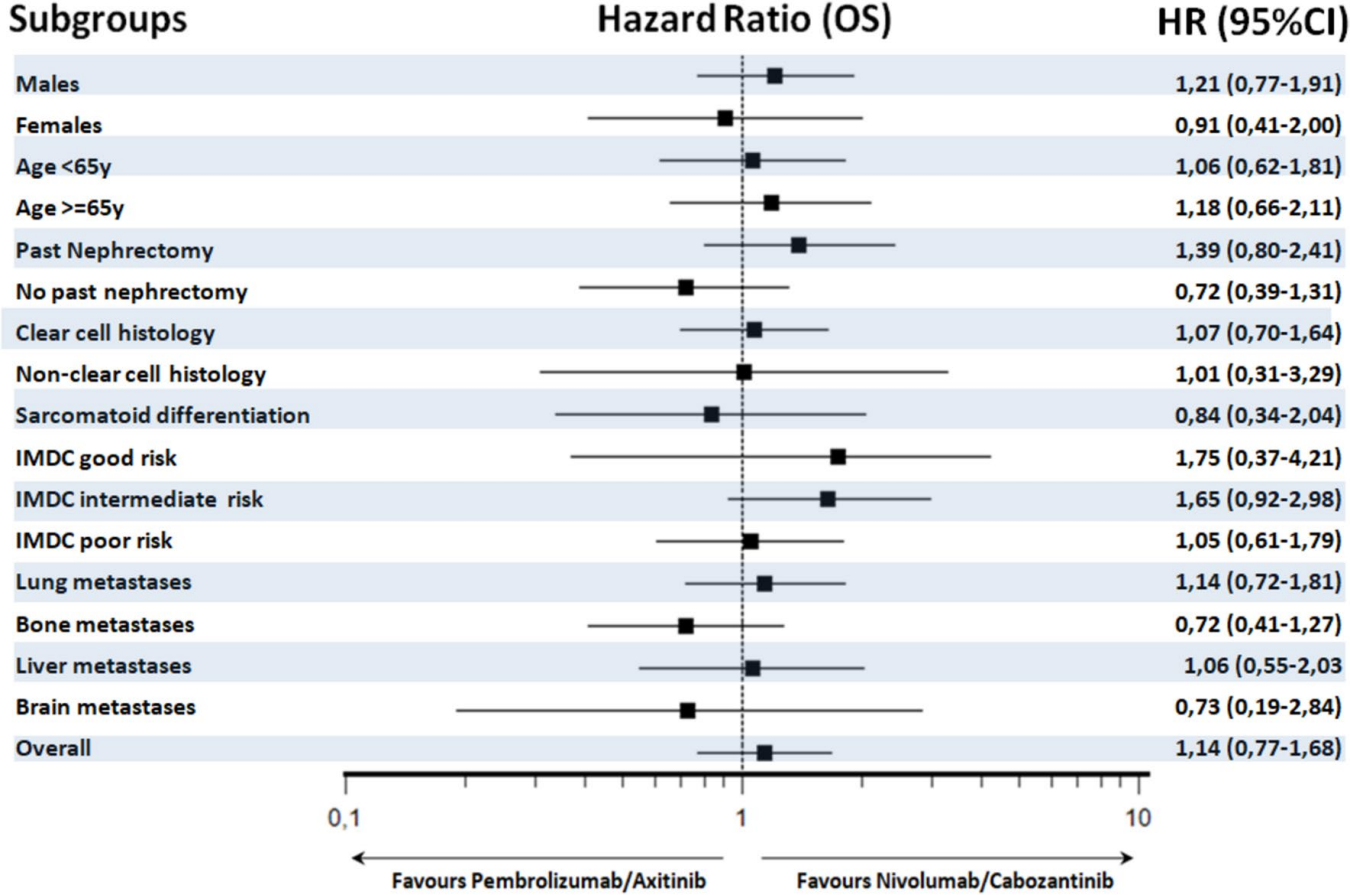
Subgroup analysis of overall survival

**Fig. 4:**
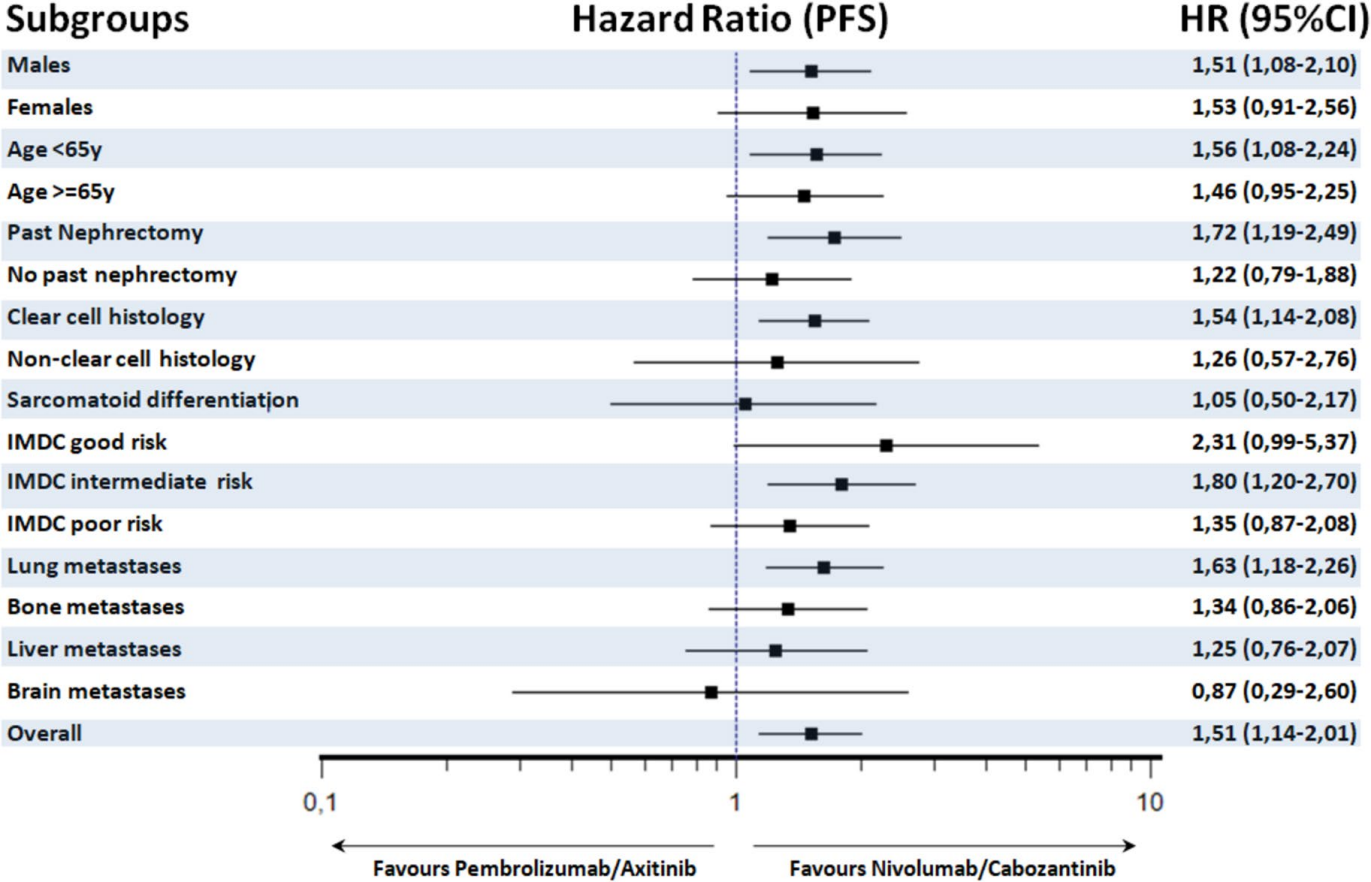
Subgroup analysis of progression-free survival

Subgroup analyses for OS and PFS are shown in Figs. 3 and 4.

### Response to first-line therapy

In the overall study population, NC yielded higher ORR as compared to PA (59% *vs.* 49%, respectively) although in the absence of a statistically significant difference; furthermore 7% CR, 52% PR, 27% SD and 14% PD were observed the former combination, as compared to 5% CR, 44% PR, 34% SD and 18% PD with PA. Notably enough, a statistically significant difference in terms of response to therapy in favor of NC was observed in patients with good risk features (Table 2).

**Table 2:** Response to therapy in RCC patients stratified by type of first-line therapy and IMDC group

Patients	Overall (%)	Pembrolizumab + Axitinib (%)	Nivolumab + Cabozantinib (%)	*p*
Overall Study Population				
Complete Response	5	5	7	.51
Partial Response	45	44	52	
Stable Disease	33	34	27	
Progressive Disease	17	18	14	
IMDC Good Risk				
Complete Response	10	7	27	< .001
Partial Response	49	49	53	
Stable Disease	40	36	20	
Progressive Disease	1	8	0	
IMDC Intermediate Risk				
Complete Response	5	5	4	.78
Partial Response	44	42	46	
Stable Disease	34	37	31	
Progressive Disease	17	16	19	
IMDC Poor Risk				
Complete Response	1	2	0	.33
Partial Response	45	43	50	
Stable Disease	24	25	19	
Progressive Disease	30	30	31	

*IMDC* International Metastatic RCC Database Consortium

### Second-line therapies

Three hundred and thirty-nine patients (45%) progressed during first-line therapy, 297 patients during PA and 42 patients with NC. Of them, 206 patients (69%) and 18 patients (43%) received second-line therapies, respectively. The median OS of the 224 patients who received second-line treatments was 31.1 months (95%CI 24.5−40.5) for PA and 40.0 months (95%CI 11.7−52.2) for NC (*P* = 0.75). Moreover, the median PFS2 was 20.7 months (95%CI 18.2−52.5) for PA and 21.8 months (95%CI 10.8−59.4) for NC (*P* = 0.48). The median OS of the 536 patients who did not receive second-line treatments was 55.7 months (95%CI 55.7−55.7) for PA and NR (95%CI NR−NR) for NC (*P* = 0.38).

### Univariate and multivariate analyses

In the overall study population, IMDC group, nephrectomy status, sarcomatoid differentiation, and the presence of bone, liver or brain metastases were correlated with both OS and PFS at multivariate analyses (Table 3). Interestingly, the choice of NC as first-line therapy was significantly associated with longer PFS at multivariate analysis (Table 3).

**Table 3:** Univariate and multivariate analyses

Overall survival	Univariate cox regression		Multivariate cox regression	
	HR (95%CI)	*p-value*	HR (95%CI)	*p* value
Gender (females vs males)	1.17 (0.84−1.63)	.34		
Age (≥ 65y vs < 65y)	1.47 (1.10−1.97)	**.009**	1.85 (1.36−2.51)	** < .001**
IMDC prognostic group	3.13 (2.47−3.97)	** < 0.001**	2.66 (2.03−3.48)	** < .001**
Nephrectomy (yes vs no)	0.40 (0.30−0.54)	** < 0.001**	0.60 (0.43−0.83)	**.002**
Histology (ccRCC vs nccRCC)	1.41 (0.97−2.04)	.06		
Sarcomatoid features (yes vs no)	1.82 (1.23−2.68)	**.02**	1,75 (1.18−2.60)	**.005**
Lung metastases (yes vs no)	1.11 (0.82−1.51)	.50		
Bone metastases (yes vs no)	2.02 (1.52–2.70)	** < .001**	1.54 (1.13−2.09)	**.006**
Liver metastases (yes vs no)	1.77 (1.29−2.42)	** < .001**	1.44 (1.03−2.01)	**.032**
Brain metastases (yes vs no)	2.05 (1.34−3.16)	**.001**	1.86 (1.17−2.96)	**.009**
Nivolumab/Cabozantinib vs Pembrolizumab/Axitinib	0.87 (0.58−1.31)	.51		

Progression-free survival	Univariate cox regression		Multivariate cox regression	
	HR (95%CI)	*p-value*	HR (95%CI)	*p-value*
Gender (females vs males)	1.25 (0.98−1.59)	.07		
Age (≥ 65y vs < 65y)	1.05 (0.84−1.31)	.68		
IMDC prognostic group	1.78 (1.52−2.10)	** < 0.001**	1.54 (1.27−1.86)	** < .001**
Nephrectomy (yes vs no)	0.51 (0.41−0.64)	** < 0.001**	0.63 (0.49−0.81)	** < .001**
Histology (ccRCC vs nccRCC)	1.40 (1.06−1.85)	**.01**	1.14 (0.85−1.52)	.38
Sarcomatoid features (yes vs no)	1.59 (1.16−2.18)	**.004**	1.53 (1.11−2.11)	**.008**
Lung metastases (yes vs no)	1.13 (0.83−1.54)	.43		
Bone metastases (yes vs no)	1.67 (1.33−2.08)	** < .001**	1.36 (1.08−1.72)	**.01**
Liver metastases (yes vs no)	1.49 (1.16−1.92)	**.002**	1.36 (1.04−1.78)	**.02**
Brain metastases (yes vs no)	1.76 (1.24−2.49)	**.001**	1.48 (1.02−2.15)	**.03**
Nivolumab/Cabozantinib vs Pembrolizumab/Axitinib	0.60 (0.43−0.84)	**.003**	0.53 (0.37−0.74)	** < .001**

*ccRCC* clear cell Renal Cell Carcinoma, *IMDC* International Metastatic RCC Database Consortium, *nccRCC* non-clear cell Renal Cell Carcinoma

## Discussion

The cornerstone of first-line therapy of mRCC has been TKI monotherapy for many years. However, a significant increase in survival rates has been reached using immune-based combinations. At present, for the first-line treatment of mRCC, international guidelines endorse the use of one of the following combinations: pembrolizumab plus axitinib or lenvatinib, and nivolumab plus cabozantinib or ipilimumab. As a whole, all these combinations, and the immune doublet in particular, proved to be less active and effective in good risk patients. PA, based on the outcomes of the Keynote-426 clinical trial, secured its position as the first approved combination of TKI and ICI [17]. In this randomized clinical trial, untreated metastatic RCC patients were randomized to receive either PA or sunitinib [6]. The PA group displayed a one-year OS rate of 89.9% alongside a PFS of 15.1 months and an ORR of 59.3%. Notably, the benefits of this combination extended across all subgroups, irrespective of programmed death-ligand 1 (PD-L1) expression or IMDC risk group. Upon an updated follow-up (with a median duration of 67.2 months), PA maintained an OS of 47.2 months and a PFS of 15.7 months. The experimental arm exhibited an ORR of 60.6%, a DCR of 83.3%, and a median duration of response of 23.6 months. The primary refractory rate was 17.0% in the PA group [18].

The most recent combination added to the armamentarium is NC, based on the positive results of the phase III trial, CheckMate 9ER [19]. This trial investigated nivolumab in combination with cabozantinib as compared to sunitinib in treatment-naïve metastatic RCC patients, irrespective of PD-L1 expression or IMDC prognostic group [7]. In the initial analysis, NC achieved a PFS of 16.6 months, with a not reached OS. The ORR was reported at 55.7% [20]. The subsequent update confirmed the superiority of NC over sunitinib, with a PFS of 16.6 months and an OS of 49.5 months within the intent-to-treat (ITT) population [21].

Notably, no head-to-head randomized clinical trials have yet compared these therapeutic approaches, while several meta-analysis have been performed, yielding conflicting results. Indeed, on one hand, Qual et al. and Riatz et al.’s meta-analyses suggested that NC may lead to the highest likelihood of delivering maximal OS [22, 23]; on the other hand, the network meta-analysis conducted by Lombardi et al. after the release of updated KEYNOTE-426 results [24] suggested that lenvatinib plus pembrolizumab had the highest probability of being the optimal treatment in terms of OS and PFS. However, among patients with sarcomatoid histology, NC demonstrated the highest rate in terms of survival outcomes.

To our knowledge, this study represents the first real-world, observational, retrospective analysis comparing PA to NC in metastatic RCC patients. Our results showed a significantly longer median PFS with NC compared to PA (27.6 *vs.* 16.2 months, respectively), a huge difference, especially considering the longer follow-up available for the PA doublet.

It’s worth noting that the PFS observed with NC is longer than that reported in the CheckMate 9ER study, while the PFS recorded with PA is in line with the Keynote-426 results. On the contrary, no differences were observed in median OS.

In our study, more patients with poor prognostic features (poor risk IMDC, bone and liver metastases) received NC, and this might partially explain why this combination yielded a clear ORR and PFS benefit, but no OS advantage in the overall population.

Subgroup analyses revealed PFS benefits for NC among intermediate risk IMDC, clear cell RCC histology, and patients with lung metastases. Moreover, in younger patients (< 65 years) and in males, NC demonstrated superior PFS, as well as in patients who had undergone nephrectomy. Response rates favored NC, especially in patients with good risk features, where CR rates almost reached 30%.

Although the use of an IO-TKI combination is recommended by most international guidelines also for the first-line treatment of IMDC good risk mRCC patients, there is an open debate about the impact of immune checkpoint inhibitors in this population, since longer follow-up of registration trials has shown declining HRs for OS in this population [25]. As a whole, our data suggest that this strategy should remain the preferred treatment. Outcomes in the second-line therapy setting showed no significant differences between treatment groups, findings consistent with previous real-world data [11]. Multivariate analysis identified NC as a significant factor associated with longer PFS. Notably, contrary to prior data, there was no evidence for additional benefit with NC in the sarcomatoid subgroup.

Differences in survival outcomes related to age and gender have been reported in the CheckMate 9ER trial, where young patients (< 65 years) and males showed a major OS benefit from NC, with no disparities in PFS. Furthermore, patients who had previously undergone nephrectomy benefited significantly in terms of OS, although not PFS, when treated with NC if compared to sunitinib. Notably, these subgroup differences were not highlighted in the Keynote-426 study.

In the first-line setting, ICI-based combination therapy significantly reduced the risk of disease progression in both male and female patients compared to sunitinib alone, with no statistically significant differences in PFS and OS between gender [26]. Likely, prior nephrectomy did not appear to influence the efficacy of ICI in metastatic RCC [27]. Conversely, the OS benefit from first-line ICI-based combinations was notably more substantial in younger patients [28].

Among the several limitations inherent in our study, the major one is related to its retrospective nature and subsequent bias, particularly considering the non-randomized allocation of patients; assignment of patients to one treatment group over the other might have been influenced by unmeasured or unaccounted-for factors, thereby potentially impacting the study outcomes. Additionally, only 20% of the patients here analyzed did receive the NC combination, the vast majority having been treated with PA, a difference which could have introduced another important bias in our analysis.

Furthermore, the proportion of patients receiving second-line therapy upon disease progression was higher among those initially treated with PA in the first line, realistically a time-lead bias related to the different timing of availability of the two combinations in clinical practice; obviously, this phenomenon could serve as a confounding factor affecting OS.

Moreover, the absence of data on safety in general, and on therapy management (dose modifications, treatment interruptions, etc.) hinders the ability to make a thorough comparison between the two-treatment regimen.

Finally, the lack of numerically adequate data relative to the third, and more recent, combination of levatinib plus pembrolizumab, prevented us to perform a more comprehensive comparison between all the different standard treatment options presently available.

Despite all the above, given the lack of data directly comparing first-line therapeutic combinations in RCC, our study offers valuable insights for treatment decisions. Indeed, it suggests a potential advantage in terms of PFS when utilizing NC as a first-line treatment, both in the overall population and among specific subgroups.

## Conclusion

Our study represents the first real-world, observational, retrospective investigation comparing PA to NC in all treatment naive metastatic RCC patients. Although inevitably biased, our results showed a significantly longer median PFS with NC compared to PA, while no differences were observed in median OS. Subgroup analyses suggested a PFS benefits for NC in various mRCC subpopulations, and an ORR benefits in the good risk population.

Additional indirect insights for treatment choice of first-line treatment between the two therapeutic regimens could be drawn from this study, although the lack of a prospective randomized comparisons between the two-treatment options (which realistically will never be performed) remains a relevant drawback of this study to take into account.

## Supplementary Information

Below is the link to the electronic supplementary material.

Supplementary file1 (TIF 175 KB)

Supplementary file2 (TIF 90 KB)

## Data Availability

No datasets were generated or analyzed during the current study.
