# Supplementary figures and images for: Pembrolizumab-axitinib versus nivolumab-cabozantinib as first-line therapy in patients with metastatic renal cell carcinoma: a retrospective real-world comparison (ARON-1)
Source: Cancer Immunol Immunother. 2025 May 27;74(7):225. doi: 10.1007/s00262-025-04043-x (PMC12116974; doi:10.1007/s00262-025-04043-x)

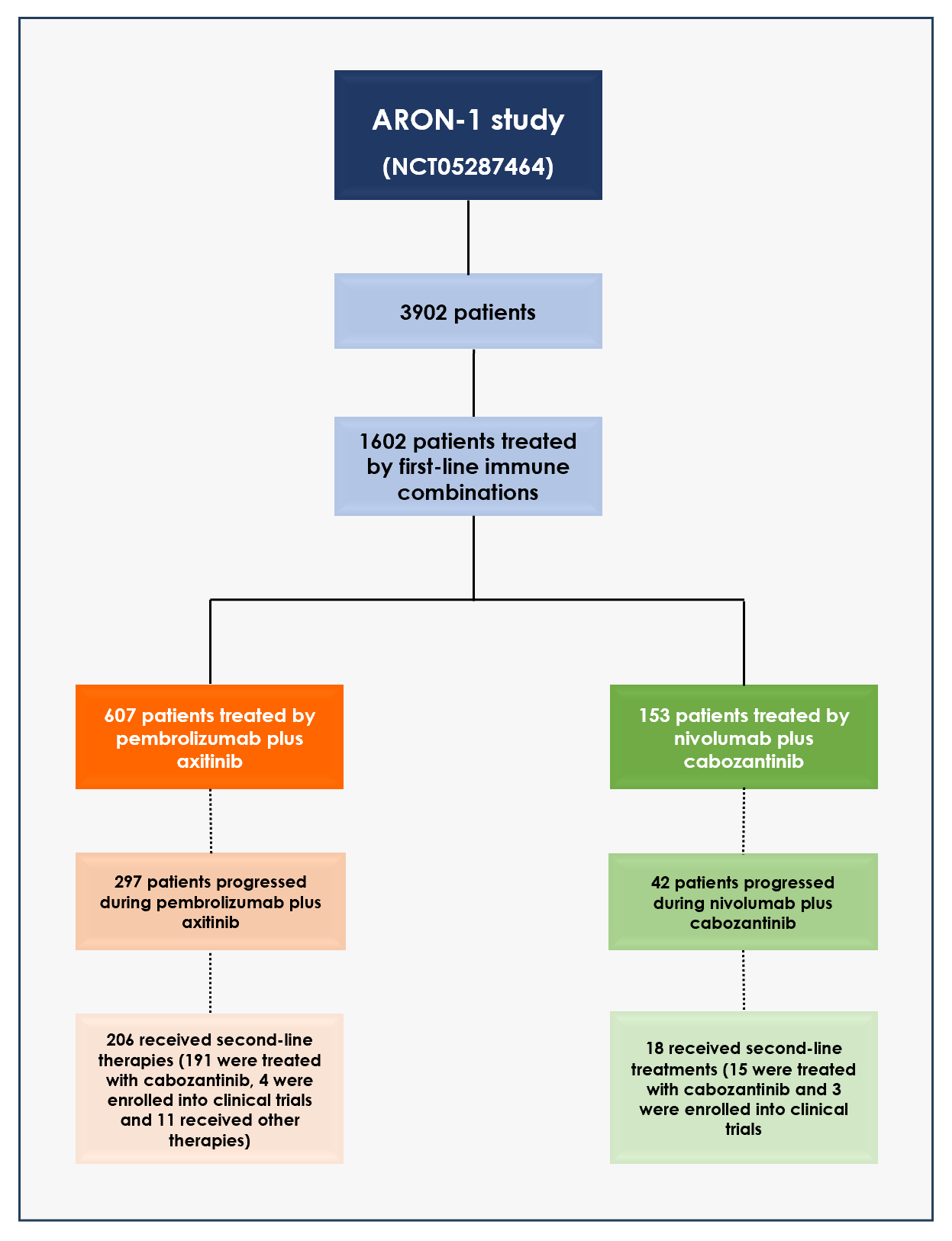

Supplement: Supplementary file 1 — Supplementary file1 (TIF 175 KB) [file 262_2025_4043_MOESM1_ESM.tif]

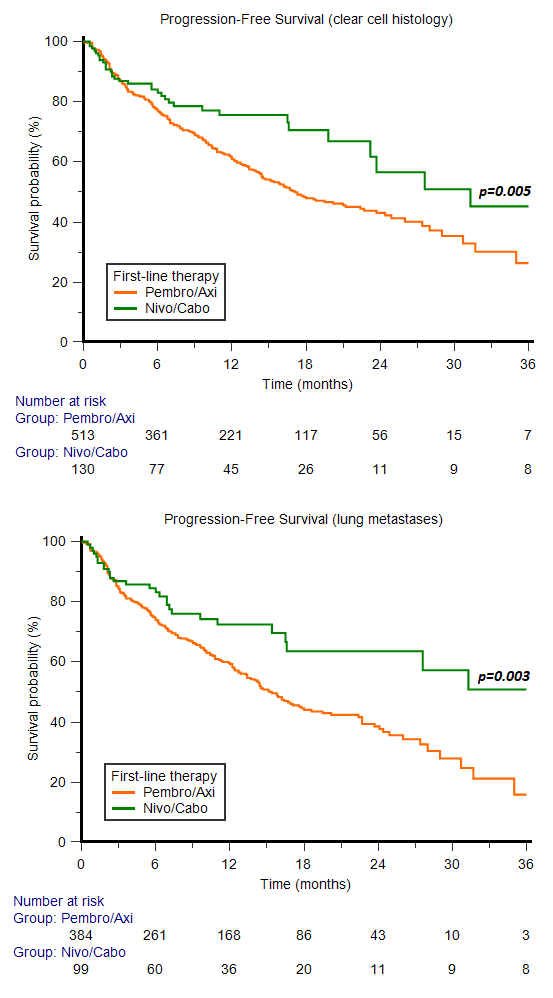

Supplement: Supplementary file 2 — Supplementary file2 (TIF 90 KB) [file 262_2025_4043_MOESM2_ESM.tif]
